# An analysis of the Sargasso Sea resource and the consequences for database composition

**DOI:** 10.1186/1471-2105-7-213

**Published:** 2006-04-19

**Authors:** Michael L Tress, Domenico Cozzetto, Anna Tramontano, Alfonso Valencia

**Affiliations:** 1 Protein Design Group, CNB-CSIC, Calle Darwin, Cantoblanco 28049 Madrid, Spain; 2 Department of Biochemical Sciences, University "La Sapienza" Rome, Italy

## Abstract

**Background:**

The environmental sequencing of the Sargasso Sea has introduced a huge new resource of genomic information. Unlike the protein sequences held in the current searchable databases, the Sargasso Sea sequences originate from a single marine environment and have been sequenced from species that are not easily obtainable by laboratory cultivation. The resource also contains very many fragments of whole protein sequences, a side effect of the shotgun sequencing method.

These sequences form a significant addendum to the current searchable databases but also present us with some intrinsic difficulties. While it is important to know whether it is possible to assign function to these sequences with the current methods and whether they will increase our capacity to explore sequence space, it is also interesting to know how current bioinformatics techniques will deal with the new sequences in the resource.

**Results:**

The Sargasso Sea sequences seem to introduce a bias that decreases the potential of current methods to propose structure and function for new proteins. In particular the high proportion of sequence fragments in the resource seems to result in poor quality multiple alignments.

**Conclusion:**

These observations suggest that the new sequences should be used with care, especially if the information is to be used in large scale analyses. On a positive note, the results may just spark improvements in computational and experimental methods to take into account the fragments generated by environmental sequencing techniques.

## Background

The environmental sequencing of the Sargasso Sea [1] has raised expectations in fields as diverse as marine ecology and energy conservation. As a result of the work a huge new resource of genomic information, comprising more than one million distinct protein sequences from an estimated 1,800 new species, has been made available to the public sequence databases.

The quantity of new protein sequences made available by this project is remarkable. At the time of their sequencing there were almost as many protein sequences in the Sargasso Sea resource as were held in the current public databases. At 90% redundancy the combination of SWISSPROT, TREMBL and TREMBLnew databases [2], for example, contained 783,110 protein sequences as of April 2004, while the Sargasso Sea resource has 643,044 sequences. The environmental genomics community has plans to gather bacteria from more of the world's oceans and from other environments, which makes the released sequences only a taste of what is to come.

While the protein sequences in the public online databases were derived from organisms from a wide range of ecosystems, the sequences from the Sargasso Sea are from a clearly differentiated marine environment. In addition, the species sequenced from the Sargasso Sea, and those that will be sequenced in similar projects in the future, are non-cultivated species, something else that sets them apart from the species whose sequences have traditionally made up the protein databanks. The details of the process for identifying genes (alignments with bacterial protein sequences were used to determine the most likely coding frames and the stop and start codons) is also likely to play a role in the relative distribution of sequences in the database.

One further difference from the sequences in the current databases is the technology used to sequence them. The Sargasso Sea sequences come from a pull of the entire DNA present in the Sargasso Sea and were sequenced using shotgun technology with low coverage. Hence there are no complete genomes present in the resource and for most of the annotated genes the species is unknown. Many reads are unassembled or partially assembled DNA fragments.

Initial analyses [3,4] have compared the functional and base composition of the sequences in the Sargasso Sea database with several other environmental resources. Here we hoped to answer a different question, how the Sargasso Sea sequences are distributed in the context of known protein families. We were interested in how the composition and structure of the new sequences influences their classification into previously known protein families, whether the new sequences complemented the sequences in the existing public databases or whether they formed distinct groups separated by discontinuities. If the new sequences were distinct, to what extent were they different to the sequences from the cultivated species in the current databases and what can they tell us about the limitations of the analyses based on those sequences?

We find however that the high proportion of sequence fragments in the resource means that it is impossible to reach any conclusions about the sequence distribution and that studies carried out with the new resource may be unduly biased by these sequence fragments. Since it is beyond doubt that environmental sequencing projects will push the numbers of protein sequences far beyond today levels, it is important to understand the effects of such large numbers of sequences from such radically different origins will have on our understanding of sequence space and what effect will this have on analysis of protein structure and function.

## Results and discussion

### Make up of Sargasso Sea protein sequence resource

**Figure 1:**
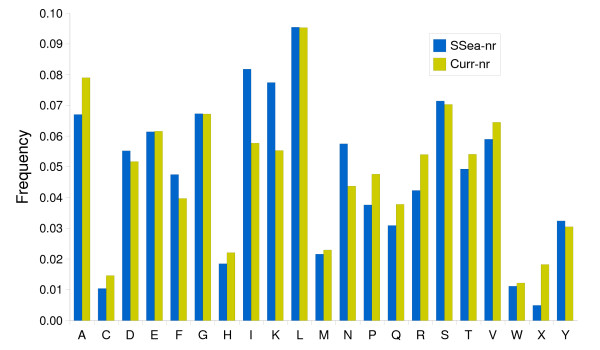
**Mean amino acid composition of the current database and the Sargasso Sea resource. **Bars in blue represent the proportion of each residue in the SSea-nr database, those in yellow show the corresponding distribution for the Curr-nr sequence database.

The sequences have a low GC-content and consequently a high isoleucine, asparagine and lysine content (40% over average) coupled with decreases in the content of certain other amino acids (see Figure 1). The isoleucine, asparagine and lysine content and the lower GC-contents of the Sargasso Sea is only comparable to a small number of other bacterial genomes, such as *Staphylococcus aureas*, *Borrelia burgdorferi*, and *Campylobacter jejuni*[5]. The relative figures for isoleucine, lysine and asparagine are 8.58, 7.56 and 5.68% for S. aureus and 8.18, 7.74 and 5.75% for the Sargasso Sea sequences. While it is surprising to find that an entire environment can have such a distinct GC-content, a recent study by Foerster et al. [4] has confirmed our findings and suggests that environment may actually shape GC-content.

**Figure 2:**
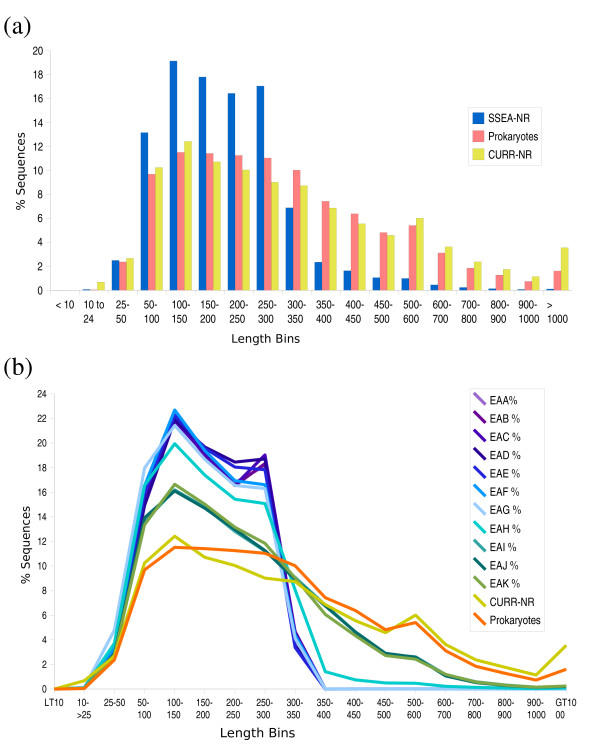
**Length distribution for the Sargasso Sea resource. **Figure 2a shows the percentages of sequences in each of 18 different length bins. Bars in blue represent the number of sequences in each bin for the SSea-nr database, those in yellow show the corresponding distribution for the Curr-nr database and those in orange show the length distribution for sequences from all completely sequenced prokaryotic genomes. The distribution shows clearly that the lengths of the Sargasso Sea sequences are much more highly concentrated at lengths between 50 and 300 residues. In figure 2b the same bins are used, but the Sargasso Sea sequences are split into their eleven constituent parts. This shows clearly that most of the fragments are to be found in sections "eaa" to "eah". The section "eaa" contained only 21,000 sequences, compared to the 100,000 sequences in the other bins.

Another very important observation is that the Sargasso Sea sequences are shorter, on average 205 residues compared to the typical 342 of the Curr-nr database. In fact many Sargasso Sea sequences are fragments of whole protein sequences and this fact has been explicitly mentioned before [6]. The influence of sequence fragments can be seen graphically when the distribution of Sargasso Sea sequence lengths is compared to those from a non-redundant database made up of SWISSPROT, TREMBL and TREMBLnew sequences (the Curr-nr database) and those from a database made up of the sequences from all completed bacterial genomes (Figure 2a).

The protein sequences in the Sargasso Sea resource are split into 11 sections by name. Of these 11 sections, 9 contain 99,999 sequences each. The first section (which has identifiers beginning with the triplet "eak") contains over 80,000 sequences and a smaller 11th section (with identifiers beginning with "eaa") has the remaining sequences. The distribution of sequence lengths within each of these sections is not identical, as can be seen in figure 2b. The figure shows that eight of the 11 sections compared ("eaa" to "eah") are composed entirely of sequences with fewer than 400 residues. While the other three sections ("eak", "eai" and "eaj") do contain sequences of greater than 400 residues, there are relative fewer full length sequences than would be expected from the sequence length distributions of the whole prokaryotic genomes, which have a significant tail of longer sequences.

### The relationship between the Sargasso Sea sequences and known protein families

BLAST [7] searches of the Sargasso Sea database failed to find a single similar sequence for 47 of the 237 query sequences. For 14 of the 47 cases, remotely related Sargasso Sea sequences could be found using PSI-BLAST when the Sargasso Sea sequences were combined with the Curr-nr sequences (the Combined-nr database), something which indicates that the standard sequences are able to occupy an intermediate position between the query sequence and Sargasso Sea sequences in a small number of cases.

However, combining the Sargasso Sea sequences with the current non-redundant databases did not appear to help in the search for remotely homologous proteins. As part of the investigation into the effects on alignments for structure prediction (see below), PSI-BLAST searches were carried out with a set of 51 query proteins (63 domains) from the homology modelling section of the CASP 4 and CASP 5 (the Critical Assessment of Techniques for Protein Structure Prediction) experiments [8]. The searches were supposed to determine whether the sequences from the Sargasso Sea (the SSea-nr database sequences) would help to detect templates that could be used in model building. PSI-BLAST profiles were first generated as per the methods section and then the Protein Data Bank (PDB) [9] was searched with the profiles. Candidate sequences that could be used as templates were discovered for 48 of the 63 domains in searches of the standard database (Curr-nr), while when the Combined-nr database was used (the database to which the SSea-nr sequences had been added) only 42 of the 48 templates could be identified. The addition of the Sargasso Sea sequences actually decreased the capacity to detect templates.

The same effect was apparent when searching for sequences belonging to known protein families. We compared those 181 query proteins for which PSI-BLAST had detected at least 5 homologous sequences from searches of each of the three sequence databases (SSea-nr, Curr-nr, and Combined-nr) and found that searches of SSea-nr database turned up fewer sequences on average (618 sequences) than searches of the Curr-nr database (807 sequences). PSI-BLAST searches of the Combined-nr database turned up fewer sequences than the two databases separately (1099 sequences), of which almost half were from SSea-nr database. The number of sequences found from the Curr-nr database, the database that included the sequences with functional and structural information, dropped to just 552.

While some small variations might be expected due to differences in E-values related to the different size of the databases being used, case by case investigations documented below made it clear that what was actually happening had little to do with database size.

In many cases searches of the Sargasso Sea and the Combined-nr databases reached a point where fewer sequences were found with successive iterations. For example, a search of the Sargasso Sea with the query 1qorA found 2740 sequences on round 2 and only 1559 on round 3. In other words the profile used to search for sequences in the 3rd round finds 1,181 fewer sequences before converging. In this case the profile has lost 40% of the sequence information. This did not happen with the corresponding searches of the Curr-nr database with this target. The same thing happens with query 2dkb (2122 sequences on round 3, 1766 on round 4) and with a number of others.

### Profiles and optimal sequences

PSI-BLAST creates multiple sequence alignments using the sequences it finds in each search iteration. The program then constructs a profile based on the frequency of amino acids at each residue position in the multiple sequence alignments and by taking into account substitution matrices. PSI-BLAST uses these profiles to search the databases on subsequent search iterations, so the information contained in the profiles is directly linked to the sequences found with each iteration. In order to investigate the odd effects of addition of the new sequences to the databases, and in particular how searches based on known families are affected we concentrated on these profiles.

Profiles are effectively a matrix formed by the 20 amino acids and the number of residue positions in the query sequence. Each residue in each position in the matrix has a probability score associated to it, a probability score that is calculated from the frequency of each residue in that position in the multiple alignment and from the replacement probabilities that come from the substitution matrix that is used.

The profile can be used to derive the so-called optimal sequence, defined as the sequence that can be obtained from the highest scoring residues in each position in the profile. The sequence reflects the conservation of each residue position and also the similarity score of the residue pairs. The profile generated from a correctly aligned set of homologous proteins should be enriched in high-scoring residues in those positions that have the most conserved amino acids in the family. In these cases the optimal sequence will be similar to the homologous proteins that have gone to make up the profile.

**Figure 3:**
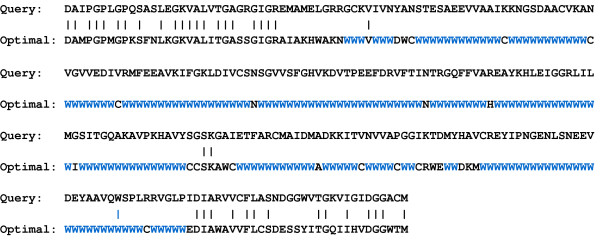
**Query sequence 1ybvA aligned with the optimal sequence drawn from the PSI-BLAST profile **The optimal sequences generated from the PSI-BLAST alignments have large stretches of tryptophan repeats that show where the profile has lost evolutionary information. Here the query sequence (above) is aligned with its optimal sequence. Repeated tryptophans in the optimal sequence are highlighted in blue.

However, if the conserved positions are not properly aligned, the optimal sequence will reflect random matching residues and, by virtue of its definition, will be dominated by residues with a high average similarity score, i.e. by rare residues such as tryptophans and, to a certain extent, cysteines (Figure 3). This is what can be observed in the optimal sequences generated from Sargasso sequences. The effect has nothing to do with the residue make up of the Sargasso Sea – the Sargasso Sea sequences have the same percentage of tryptophans and cysteines as the sequences in the current databases and actually have less low complexity regions than the current databases. On closer inspection these rare residue repeats were also found in the optimal sequences of PSI-BLAST profiles generated from other databases – however, they were found much more frequently in profiles generated from searches of the SSea-nr and Combined-nr databases.

**Figure 4:**
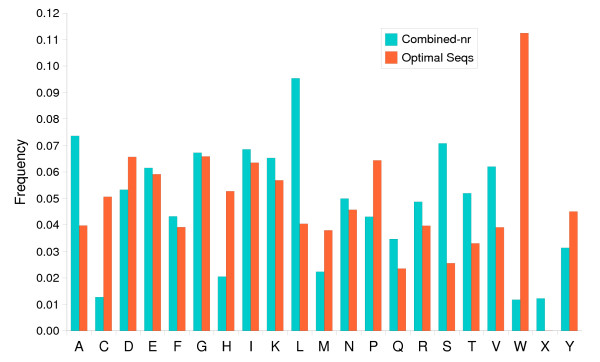
**Amino acid residue make-up of the optimal sequences. ** Bars in turqoise represent the proportion of each amino acid residue in the Combined-nr database from which the PSI-BLAST profiles were built. Bars in orange also show the proportion of each amino acid, this time from the optimal sequences generated from the same profiles.

That the optimal sequences extracted from the PSI-BLAST matrices contain high proportions of tryptophans and cysteines can be seen clearly in Figure 4. Here we compare the proportions of each residue in the optimal sequences generated from PSI-BLAST searches of the Combined-nr database with the background levels of each residue in the sequences of the Sargasso Sea and Current databases that make up the Combined-nr database. There are ten times as many tryptophan residues in the optimal sequences as in the database sequences and cysteine is represented five times more in the optimal sequences than in the databases.

### Alignment conservation (entropy)

**Figure 5:**
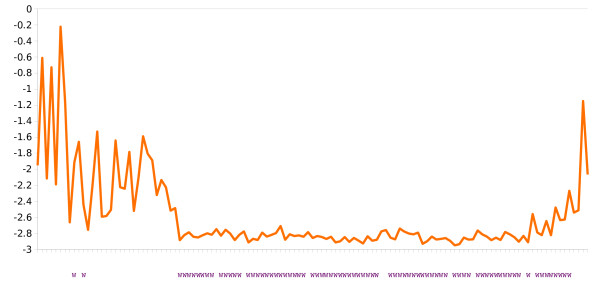
**A plot of residue entropy against the optimal sequence for CASP target T0171. **Here the relative entropy is plotted against the optimal sequence of query T0171. Relative entropy is calculated from the alignments from the final iteration of the PSI-BLAST search of the Combined-nr database. The optimal sequence is drawn from the PSI-BLAST profile of the same target. Notice that the entropy flattens out and that this region of low entropy and low conservation coincides with the long stretch of repeated tryptophans in the optimal sequence. Entropy is shown on log scale.

**Figure 6:**
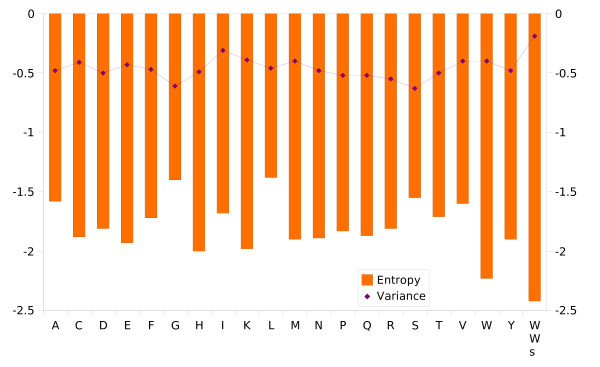
**A plot of entropy versus optimal sequence residue. **Bars in orange show the mean relative entropy for each of the 20 amino acids. Entropy is shown on log scale. Amino acid type for each residue position was determined from the optimal sequences generated from the PSI-BLAST profiles and came from searches of the Combined-nr. The bar marked "WW" refers to tryptophans in repeats. The purple line shows the variance of the relative entropy. Positions with repeat tryptophans clearly have the lowest mean entropy and the lowest variance.

The optimal sequences are generated from the PSI-BLAST profiles for each target, while the profiles are calculated directly from the PSI-BLAST alignments. It is possible to measure the conservation of residue positions in an alignment using residue entropy. We calculated the residue entropy for all 237 of the PSI-BLAST multiple alignments generated from searches of the Combined-nr database (which contains both Sargasso Sea and current database sequences) and plotted entropy directly against each of the optimal sequences for each of the 237 target sequences (we show an example in Figure 5). We also plotted entropy against the residue type of the optimal sequences drawn from the profiles (Figure 6).

The plot of entropy versus optimal sequence residue (Figure 6) clearly correlates tryptophans in the optimal sequences with low entropy and therefore with poor residue conservation. Tryptophan is the most frequent residue in the optimal sequences; it has the lowest entropy and the lowest variance around the mean of all the residues. Not only that, but repeated tryptophan residues have even less entropy and very little variation in entropy score, showing that repeated tryptophans always mark residues with little or no evolutionary information.

One more example of the relationship between entropy and optimal sequence is shown in the plot of entropy against the optimal sequence of target T0171 in Figure 5. While much of the optimal sequence is characterized by a series of jagged peaks and troughs, representing the variable levels of conservation at each position, the part of the optimal sequence that is a long string of tryptophans essentially flat-lines, showing that all conservation has disappeared from these residues.

The regions of repeated rare residues in the optimal sequences are clearly symptomatic of those regions of low entropy and low conservation that are devoid of all evolutionary information. These repeated residues are most often tryptophan. We chose to use a scoring scheme based on the repeated rare residues (Profile Discriminatory Quality, see below) in order to make comparisons, because this score better highlighted the clear differences between the different databases used in the study.

### Profile discriminatory quality

We calculated the discriminatory quality of the three databases used for the query proteins searches as per the methods section. Discriminatory quality was the percentage of the optimal sequence that was not made up of tryptophan or cysteine repeats. If the optimal sequences are free of cysteines and tryptophans, the profile discriminatory quality will be 100. The discriminatory quality of the current databases (Curr-nr) is considerably higher than the Sargasso Sea database. The discriminatory quality score of profiles generated from the Curr-nr database is 93.69 over all query sequences, compared to 88.49 for the SSea-nr database. Therefore searches against the SSea-nr database turn up optimal sequences with almost twice as many tryptophans and cysteines as searches against the current databases.

The effect of combining the two sequence databases is to make the discriminatory quality of the profiles substantially worse – the Combined-nr database has a discriminatory quality score of just 85.22 over all query sequences. Profile Discriminatory Quality was calculated from profiles generated for all 237 target sequences so the SSea-nr and Combined-nr scores include those PSI-BLAST searches which found no sequences and therefore will have had discriminatory quality scores approaching 100.

Given the strange composition of the Sargasso Sea sequences in terms of fragments (fig 2a) it is quite possible that the presence of fragments is behind the results obtained with the database searches.

In order to find out if this is the case and what other reasons might be causing the odd behaviour of the Sargasso Sea sequences, we created three more databases that we could use for comparison. Two of the databases were created in order to eliminate as many fragments as possible. First to investigate the effects of fragments on the profiles we created a combined 90% redundant database from the Curr-nr sequences and sections eak, eaj and eai of the Sargasso Sea (Combined_itok). These three sections have a length distribution that is much more similar to that of the Curr-nr and the combined prokaryote databases (see Figure 2). This version of the database contained 1,025,174 sequences

While many of the fragments from the Sargasso Sea were eliminated while creating the Combined_itok database, it was clear from Fig 2b that there are still a number of fragments in sections eak, eaj and eai of the Sargasso Sea resource. We attempted to remove as many fragments as possible, this time by creating a Sargasso Sea resource with a minimum sequence size of 250 residues. Although not all the fragments will have been removed, the smallest fragments will have been taken out. The non-redundant database created in this way (Combined_GT250) had 1,053,952 sequences, approximately the same size as the Combined_itok database (1,025,174 sequences).

As a comparison and in order to eliminate the effect of database size we generated an updated version of the Curr-nr database, this time with sequences from the April 10, 2005 version of the combined SWISSPROT, TREMBL and TREMBLnew databases. This database contained no sequences at all from the Sargasso Sea resource and had 1,005,858 sequences, almost the same size as the two databases created above.

The profile discriminatory quality for these two new databases was measured as in the methods section. The results are shown in Table 1. It seems that increasing size of the search database makes little difference to discriminatory quality. What does make a difference to the discriminatory quality is the fragment content.

The mean discriminatory quality of the profiles generated from the Curr-nr (April 2005) and Curr-nr (April 2004) databases are almost identical despite the difference in database size. Despite the fact that the Curr-nr (April 2005) and Combined_itok are practically the same size, the mean discriminatory quality of the profiles generated from the latter are considerably worse. This confirms that the strange effects that are being seen in the Combined_itok and Combined-nr databases are not simply the result of adding new sequences to the existing databases.

While reducing the number of fragments by using the better quality sequences from sections eaj, eai and eak does have the effect of improving the mean discriminatory quality of the profiles, the improvement is by less than one point. However, removing those fragments with fewer than 250 residues improves the discriminatory quality score by 6.5 points compared to the Combined-nr database and by more than 5.5 points combined to the Combined_itok database.

One other difference between the results from these two databases was that there were more Curr-nr sequences found from searches of the fragment-poor Combined_GT250 database (778 sequences on average) than from the fragment-rich Combined_itok database (631 sequences). The fact that more Curr.-nr sequences were found with the higher quality Combined_GT250 database suggests that removing most of the fragments increases the searching power of PSI-BLAST.

The profile discriminatory score for the Combined_GT250 database is still not as good as that of the Curr-nr (April 2005) database, but this difference is almost certainly due to the fact that the Combined_GT250 database still contained fragments, fragments that were greater than 250 residues in length and that affected profiles generated for the longest of the query sequences.

As a further test we also created our own fragmentised database with the April 2005 version of the Curr-nr database. All sequences were split randomly once and the largest part of the sequence retained for the database. Searches with the 237 query sequences of this database gave profile discriminatory scores of 89.1, compared to the 93.36 of the non-fragmented database, confirmation that a simple fragmentation of the current databases was enough to recreate much of the deleterious effects of the Sargasso Sea sequences.

### An example: the Ftsa family

We looked at one family in particular, the Ftsa family. Ftsa is essential for bacterial cell division. We took the *Thermotoga Maritima *Ftsa sequence from the solved PDB structure 1ef4A and used it as the query in a PSI-BLAST search of the Combined-nr database. 1ef4A has 419 residues. There are three clear ATP-binding motifs, one in the N-terminal, one in the centre of the sequence and one at the C-terminal end of the sequence [10].

The sequences found in each round were aligned using CLUSTALW [11]. The results were instructive even in the first iteration (effectively just a BLAST search). BLAST found 165 sequences from the combined database, 74 from the Curr-nr database and 91 from the SSea-nr database. The alignment showed that all the sequences bar one from sections "eaa" to "eah" of the Sargasso Sea database were fragments that were not long enough to align all three binding motifs. In fact even 20 of the 42 sequences detected from sections "eak" to "eai" of the Sargasso Sea database also turned out to be fragments and not long enough to align all three motifs. None of the Curr-nr sequences were fragments.

Interestingly one of the 22 whole protein sequences from the Sargasso Sea found by the BLAST search had mutations in each of the three motifs that were not apparent in the sequences from the Curr-nr database, a second whole sequence had mutations in two of the motifs and a number of the fragments from the SSea-nr database had unique and distinct mutations at the C-terminal motif. This suggested that there might indeed be sequence variations in the families present in the Sargasso Sea resource, although clearly this effect was from a single example and on a large scale any such effects were being drowned out by the poor alignments and the high fragment content.

In the second round CLUSTALW was no longer able to produce a good alignment of the 776 sequences found. While the first motif is relatively well aligned, the central motif is not at all aligned. The alignment from MUSCLE [12] is somewhat better, though far from perfect. The central motif is well conserved in this alignment. The N-terminal and C-terminal motifs are not well conserved, though more than half the sequences found in the second round are too short to have both the N-terminal motif and the C-terminal motif. One fragment has only 32 residues.

The results from the second round show how the fragments invade the profile and begin to destroy the discriminatory quality. After 4 rounds the N-terminal and C-terminal motifs are still recognisable in the optimal sequence calculated from the PSI-BLAST profile, but the central motif has disappeared.

### Additional features of the Sargasso Sea resource

During the sequence analysis we detected a number of interesting differences with the standard behaviour of sequence families from the current databases. Here we describe some of them, with particular emphasis on the influence of the high proportion of fragments in the resource. Differences may be related to differences in family distributions or may simply be due to the influence of the anomalous sequence size distribution.

### Regions of low complexity

We ran the low complexity detection program SEG [13] for all the sequences in both the Curr-nr and SSea-nr databases in order to detect regions of low complexity in the sequences that might be biasing the PSI-BLAST searches. SEG finds that in fact the Sargasso Sea sequences have proportionally more complexity than the sequences in the current databases (Curr-nr). 5.7% of the Sargasso Sea sequences are masked by SEG, compared to almost 8% of the sequences from Curr-nr (see Figure 1). A database composed of all prokaryotic sequences from complete genomes had just 6.2% of residues in SEG-defined low complexity regions, suggesting that the complexity of the Sargasso Sea sequences was in line with what would be expected. PSI-BLAST searches with the query sequences were carried out both with SEG on and off. It made little difference to final result.

### Sequence clustering

**Figure 7:**
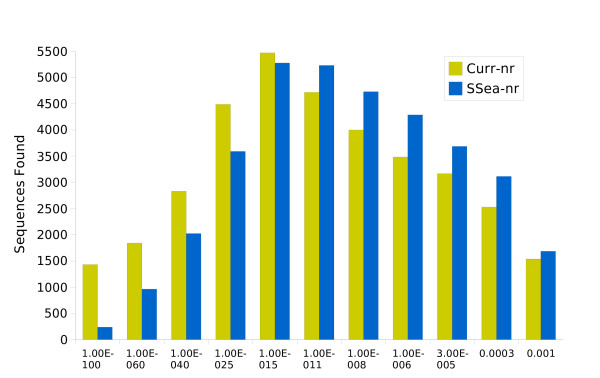
**Distribution of BLAST e-values. **Here the sequences all come from BLAST searches of the Combined-nr database. Here BLAST runs with the 237 target sequences finds as many related Sargasso Sea sequences as sequences from the current database. The distribution of the sequences is a difference between the two sequence resources though, sequences from the Sargasso Sea databases are found with higher E-values on the whole, something that is probably a side effect of the high proportion of fragments in the resource.

To assess the distribution of the Sargasso Sea sequences in relation to the rest of the known sequences, we collected all sequences found from the BLAST searches of the Combined-nr database (as above). Fig. 7 shows the results of the distribution of the E-values from the BLAST searches. These scores are a measure of the similarity between the detected sequence and the query.

Even though the Combined-nr database contained substantially more Curr-nr sequences than SSea-nr sequences, BLAST detected as many Sargasso Sea sequences as Curr-nr sequences. However, the Sargasso Sea sequences were found with substantially higher E-values. While the shapes of the two distributions are similar, the Sargasso Sea sequence distribution is shifted relative to the Curr-nr sequence distribution and it has a higher mean E-value (lower level of sequence similarity with the target sequence). If there were no length bias in the Sargasso Sea database, this behaviour would indicate that the Sargasso Sea sequences were more divergent. However, the higher E-values of the Sargasso Sea sequences is also likely to be due to the amount of sequence fragments in the databases since in BLAST the shorter the alignment, the higher the E-values in general. This shows too that even the results of BLAST searches with Sargasso Sea resource should be treated with extreme caution.

### Redundancy

To investigate further the different sequence distribution we created a sequence database from all the bacterial and archaea sequences in the SWISSPROT and TREMBL databases. As a comparison we created a second database of a similar size from sequences from groups "ead" to "eak" of the Sargasso Sea. Both databases contained approximately 780,000 sequences. We used cd-hit [14] to create non-redundant databases for the two at 90, 80, 70, 60 and 50%. The results are shown in Table 2.

It is clear that the Sargasso Sea sequences have more redundancy. This might perhaps not be surprising given their dependence on a very unique ecosystem and might it would be easy to leap to wrong biological conclusions, but again it might be wrong to interpret the results this way since this pattern would also be typical of a database composed of fragments of sequences.

### Sequences in homology modelling

The capacity to build models by homology is one of the techniques that have improved over recent years, in part due to the expansion of the sequence databases. The different organisation of the Sargasso Sea sequences with respect to the previously known databases might affect this capacity. To assess this question we compared the accuracy of the alignments that could be obtained with and without the Sargasso Sea sequences.

The addition of the new sequences adversely affected the quality of the pair-wise target-template alignments in 12 of the 32 cases we tested. In these cases the difference in the number of correctly aligned residues in the pairwise alignments implied by the multiple sequence alignment was an average of 17%. In 11 cases the differences were small (no more than 1%), and in just seven cases the alignment shows a modest improvement (an average of 10%), demonstrating again that far from improving the quality of the models, the Sargasso Sea sequences have a tendency to decrease model quality. Again this could be a consequence of sequence fragments.

## Conclusion

The sequences from the Sargasso Sea differ markedly from those currently in the databases, only 11,700 sequences (under 2%) of the 90% redundant Sargasso Sea database overlapped at 90% identity with the equivalent sequences from the current databases. In addition the new sequences have a much higher isoleucine, asparagine and lysine content and are considerably shorter on average than the sequences currently in the databases. This last observation is due to the sequence fragments from incomplete ORFs that are found in all sections of the Sargasso Sea resource.

The Sargasso Sea sequences form the first large set of environmental sequences released to the databases and it is therefore interesting to investigate the consequences of adding a great number of sequences from a radically different environment to the protein families in the current databases. For example, some of these new environmental sequences may well occupy distinct and differentiated regions of sequence space at the periphery of the previously known protein families, or may be effective at populating sparsely-populated protein sequence space.

From a practical point of view, more sequences in the databases ought to lead to more powerful automatic tools for sequence searching, creating multiple alignments and predicting function by linking clusters in sequence space. In particular it is a commonly held belief that the growth of sequence databases has increased and will continue to increase our capacity to define protein families [15], propose new functions [16,17], predict binding sites [18], predict secondary structure [19] and derive models by homology [8].

We analysed whether the new sequences fulfilled their promise and to what extent they could be assigned to known families from the standard databases. However, we found that the high proportion of sequence fragments in the resource made it impossible to reach any conclusions about the sequence distribution. In addition the new sequences result in more profile drift, a decrease in the quality of pairwise and multiple alignments, more difficulty in detecting homologues and defining families and conserved functional regions.

Our results show that PSI-BLAST multiple alignments built from these sequences tend to have large, poorly aligned regions with little conservation and low entropy. These "dead" zones of poor conservation and low entropy are characterised by repeated rare residues in the optimal sequences drawn from the profiles. The dead zones indicate where profiles have lost evolutionary information and search power – in fact those profiles that contained large dead zones also often found fewer sequences with successive PSI-BLAST iterations.

PSI-BLAST has many well documented flaws [20], none of which were found to have had any bearing on the overall results. The strange results are almost certainly an example of severe database contamination. The poor quality of the alignments generated from the Sargasso Sea sequences were universal, the other two multiple methods used in this study, CLUSTALW and MUSCLE, also had great difficulty in aligning related Sargasso Sea sequences. Nor are hidden Markov model methods much more successful at generating profiles with Sargasso Sea sequences (A. Rojas, personal communication).

We have shown conclusively that the peculiar behaviour of the Sargasso Sea sequences in this study is caused by the high proportion of sequence fragments. The fragments adversely affect the building of multiple sequence alignments and profiles. The results show that even where sequences can be clustered into sequence families recognisable from PSI-BLAST searches, the fragmentary nature of the new sequences often distorts the multiple alignments to such an extent that family characteristics are lost.

Chen and Pachter [21] have recently highlighted the problems of including partial, fragmented sequences from environmental sequence projects in phylogenetic analyses and in multiple sequence alignments. They describe the problem as an extreme case of the missing data problem [22]. This is almost certainly what is happening here as well. Since almost all multiple sequence alignment methods penalise terminal gaps, they are not good at aligning sequences if there is a high proportion of partial, fragmented sequences in the sequences to be aligned.

### The practical consequence of the Sargasso Sea sequences for bioinformatics tools

As we have shown here, the quality of the sequences in the Sargasso Sea resource means that it is difficult to carry out large scale investigations into whether these sequences represent a discontinuity in the previously known protein sequence space, or whether our knowledge is biased towards the small corner of the ecosphere we know about.

When first released these environmental sequences were included in many of the public searchable databases, and for a time results from the main publicly available BLAST servers were tainted by the sequences. They have since been removed from all the main web-based BLAST servers [23]. These results justify the decision to remove them on the grounds that the fragments were distorting the searches and the profiles.

The expansion represented by these environmental sequences exposes certain limitations in the current techniques. If researchers are to make use of the new wealth of environmental sequences, how will they deal with the problems caused by the high proportion of sequence fragments if the new sequences are of such poor quality? This is an emerging problem, not only because of the number of environmental sequencing projects currently underway, but also because sequence fragments are being deposited directly into Uniprot by gene annotation projects. Even though they are in smaller number, these sequence fragments are not benign and a number of them have already appeared in expert databases such as Pfam [24].

The hope is that these new sequences will push us to improve bioinformatics tools, possibly by developing methods better suited to deal with large numbers of incomplete sequences. Simple, makeshift solutions include filtering databases prior to their use or allowing users to put a length filter on the sequences included in multiple alignments. Meanwhile environmental sequences should be treated with care.

### The quality of the sequences and possible biological conclusion

The Sargasso Sea sequences are from a range of species subject to the same environmental pressure. This has led researchers to investigate whether there are differences from the current databases. For example, Meyer [6] used iron-sulphur proteins to suggest that the Sargasso Sea resource showed that microbial diversity has been underestimated by an order of magnitude. While the distribution of the sequences in the Sargasso Sea resource and those in the current databases may indeed be different, the results from this study suggest that additional work may have to be considered before any secure conclusions can be made.

Indeed the same is true about any study where Sargasso Sea sequences are used in database searches or multiple alignment methods. For example, while one interpretation of the E-value distribution we found in Figure 7 might be that there is true biological divergence of the sequences, the most likely explanation is that the fragment content of the Sargasso Sea resource is the cause of the higher E-values. In general, the shorter the alignments in BLAST the higher the E-values, so the E-values for the Sargasso Sea sequences must be greater simply because they are fragments. If the E-values of the partial sequences are higher than for the full sequences, then BLAST will automatically find less homologues, and so even BLAST results should be treated with care when the Sargasso Sea resource is used.

Recently there have been a rash of studies that have used Sargasso Sea sequences in comparisons using BLAST or phylogenetic profiles based on alignments [for example [3,25-27]]. While the results of these studies are of great interest, the fact that the Sargasso Sea fragments introduce a bias into such studies may need to be taken into account.

Despite these reservations we did observe interesting deviations from of the behaviour of normal families in isolated examples that might indicate that there are differences in the distribution of sequence families in the Sargasso Sea resource, small differences that are being masked by the poor quality of the Sargasso Sea sequences. Given the masking effect of the sequence fragments, it is difficult to tell to what extent these small changes are a result of the unique evolutionary pressures on the Sargasso Sea sequences and to what extent they might be due to errors resulting from the low coverage depth of the shotgun sequencing techniques used to sequence the Sargasso Sea sequences.

In the future the sequences from new environmental genomics initiatives may still provide us with invaluable insights into some of the key issues in evolution. In particular, the flooding of the databases with sequences from environmental sequencing projects may impact on key predictions for the total number of families and folds [28-30] and the number of structures needed to cover the sequence space by structural genomics efforts [31,32]. Revisiting these predictions in the light of the sequences from the environmental sequences may make us more aware of where we have reached in our efforts to describe global protein sequence space.

## Methods

### Search databases

The Sargasso Sea database was built from sequences culled from the whole genome shotgun sequencing of the Sargasso Sea from the GenBank database [33]. A 90% redundant database was created from these sequences with the clustering program cd-hit. There were just over a million sequences in the original resource deposited in GenBank and the non-redundant Sargasso Sea database (SSea-nr) contained 643,044 sequences.

There is a small fraction of the Sargasso Sea sequences, fewer than 100 sequences, that contain a non-standard amino acid (marked as X in the sequence), in every case as a result of a translation from the base "N" (any). All these 100 sequences appear in section 6 of the 17 separate environmental sequence files in GenBank, clustered in 4 close groups.

A local non-redundant database was built from the sequences stored in the SWISSPROT, TREMBL, and TREMBLnew databases as of April 2004, the date of publication of the new sequences. This database was also clustered at 90% redundancy. The resulting non-redundant database (Curr-nr) contained 783,110 protein sequences.

A third non-redundant database was built by amalgamating the two non-redundant databases. This combined database (Combined-nr) had 1,414,454 sequences at 90% identity after clustering with cd-hit. Only 11,700 sequences (0.82% of the whole database) were removed by cd-hit during the process.

### Query sequences

For the experiments involving sequence search methods we needed target sequences. We took 87 query sequences from CASP experiments 5 [34] and 6 [35] and selected 150 more query sequences from the PDB. These PDB sequences had been used in a previous study [18] because each of them had a remotely homologous PDB template that contained site residue information. A total set of 237 query sequences were used in the study.

### Creating the profiles for scoring the alignments

PSI-BLAST sequence profiles were generated from all three non-redundant databases for each of the 237 query sequences. The profiles were generated by running PSI-BLAST for four iterations and with the default options. The profiles were used to deduce the optimal sequences for each of the target sequences and each of the databases.

### Profile discriminatory quality

In a number of cases PSI-BLAST actually started to find fewer sequences with successive iterations of the databases. We assessed the profiles generated by PSI-BLAST for these sequences and the optimal sequences extracted from the PSI-BLAST multiple alignments. We found that the optimal sequences in these cases were characterised by their low complexity and by very high proportions of tryptophans and cysteines. High proportions of tryptophans and cysteines in the optimal sequences are a side effect of the loss of discriminatory quality in sequence profiles.

We used the quantity of cysteines and tryptophans in the optimal sequences from profile generated by PSI-BLAST in order to generate a measure of the discriminatory quality of each profile. Profile discriminatory quality here is defined as:

100 - (W + C-wb-cb)

where W is the percentage of tryptophans in the optimal sequences, C the percentage of cysteines in the optimal sequences and wb and wc the background percentages of cysteines and tryptophans in the sequence database that PSI-BLAST used to build the profile. If the discriminatory quality of the profiles were perfect there would be no more cysteines and tryptophans in the optimal sequences than in the sequence databases and the profile discriminatory quality would be 100.

### Sargasso Sea sequences in comparative modelling

The Sargasso Sea resource was also used to create alignments for the purposes of building 3D structural models. 31 domains from 27 CASP 4 [36] and CASP 5 [8] comparative modelling targets from a previous study [37] were used for the comparison. The targets were chosen because they were targets for which PSI-BLAST had been able to identify a structural template at the time of the CASP experiments. The best template for each of the considered CASP 4 and CASP 5 targets was defined as the protein with the highest structural similarity with the target structure according to the LGA structural alignment method [38].

Sequences were collected from both the SSea-nr database and the non-redundant databases frozen at the time of the two CASP experiments using the default options of PSI-BLAST and five iterations.

Sequences were collected that had a percentage sequence identity with the target sequence that was equal or higher to that of the best template and CLUSTALW was used to create multiple alignments, first with the sequences found from the search of the NR databases frozen at the time (the CASP-set of sequences) and then with the CASP-set sequences added to those sequences found from the search of SSea-nr (this set of sequences was called the CASP-SS-set). The pair-wise alignments between target and template implied by the multiple sequence alignment were extracted and compared with the structural alignment. Correctness of the target-template sequence alignments was computed with respect to the LGA structural alignment of the pair.

## Authors' contributions

MLT conceived of the study, designed the work, carried out the analysis and interpretation of the data and drafted the manuscript. AT and DC designed and carried out the homology modelling section. AT also helped draft the manuscript. AV participated in the design and coordination of the project, was involved in the interpretation of the data and helped to draft the manuscript.

## Figures and Tables

**Table 1:** Profile discriminatory quality (PDQ) for profiles generated from searches of six databases Results are compared for 6 databases, the combined database with all the Sargasso Sea sequences (*Combined-nr*), the public databases at the time of deposition of the sequences (*Curr-nr (April 2004)*), the same databases a year later (*Curr-nr (April 2005)*) and three databases of similar size (i) the current databases and sections eai, eaj and eak of the Sargasso Sea resource (*Combined_itok*), (ii) the current databases and sequences from the Sargasso Sea of length 250 or greater (*Combined_GT250) *and *(iii) *a fragmentised version of the April 2005 databases *(Curr-nr (April 2005) Frags)*. Profiles were generated for all 237 target sequences and profile discriminatory quality is described in the methods section. The size of each database in thousands of sequences is also shown. The table shows that profiles generated from databases with a high proportion of fragments have less discriminatory power.

***DBs with Many Fragments***	**DB Size**	**PDQ**	***DBs with Few Fragments***	**DB Size**	**PDQ**
**Combined-nr**	1414 K	*85.22*	**Curr-nr (April 2004)**	783 K	*93.69*
**Combined_itok**	1025 K	*86.17*	**Combined_GT250**	1054 K	*91.74*
**Curr-nr (April 2005) Frags**	1006 K	*89.1*	**Curr-nr (April 2005)**	1006 K	*93.36*

**Table 2:** Clustering for Redundancy with the Sargasso Sea Sequences The number of sequences in each database at different redundancy levels. *Sargasso *comprised sections ead, eae, eaf, eag, eah, eai and eak of the Sargasso Sea resource, *BactArch *was a combination of bacterial and archaea sequences from the SWISS-PROT and TREMBL databases.

	***100%***	***90%***	***80%***	***70%***	***60%***	***50%***
*Sargasso*	*780756*	*509450*	*394592*	*310768*	*245027*	*188241*
*BactArch*	*761237*	*535059*	*485811*	*434773*	*379386*	*318309*
